# New structural insights into the multifunctional influenza A matrix protein 1

**DOI:** 10.1002/1873-3468.14194

**Published:** 2021-10-02

**Authors:** Julia Peukes, Xiaoli Xiong, John A. G. Briggs

**Affiliations:** ^1^ Structural Studies Division Medical Research Council Laboratory of Molecular Biology Cambridge UK; ^2^ California Institute for Quantitative Biosciences (QB3) University of California Berkeley CA USA; ^3^ Bioland Laboratory (Guangzhou Regenerative Medicine and Health – Guangdong Laboratory) Guangzhou Institutes of Biomedicine and Health Chinese Academy of Sciences Guangzhou China; ^4^ Department of Cell and Virus Structure Max Planck Institute of Biochemistry Martinsried Germany

**Keywords:** cryo‐electron tomography, enveloped virus, helical reconstruction, influenza virus, M1, matrix proteins, protein oligomerization, subtomogram averaging, virus assembly

## Abstract

Influenza A virus matrix protein 1 (M1) is the most abundant protein within virions and functions at multiple steps of the virus life cycle, including nuclear RNA export, virus particle assembly, and virus disassembly. Two recent publications have presented the first structures of full‐length M1 and show that it assembles filaments *in vitro via* an interface between the N‐ and C‐terminal domains of adjacent monomers. These filaments were found to be similar to those that form the endoskeleton of assembled virions. The structures provide a molecular basis to understand the functions of M1 during the virus life cycle. Here, we compare and discuss the two structures, and explore their implications for the mechanisms by which the multifunctional M1 protein can mediate virus assembly, interact with viral ribonucleoproteins and act during infection of a new cell.

## Graphical abstract

Influenza A virus matrix protein 1 (M1) forms an endoskeleton underneath the membrane envelope and functions at multiple steps of the virus life cycle, including nuclear export, virus assembly and entry. Two recent publications presented the first structures of full‐length M1 protein in vitro and within the virus, providing a molecular basis to understand the multiple functions of M1.

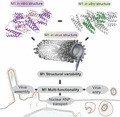

## Abbreviations

**cryoET**, cryo‐electron tomography

**CTD**, C‐terminal domain

**HA**, haemagglutinin

**M1**, matrix protein 1

**M2**, matrix protein 2

**NA**, neuraminidase

**NLS**, nuclear localization signal

**NP**, nucleoprotein

**NTD**, N‐terminal domain

**TNPO1**, transport factor transportin‐1

**VLPs**, virus‐like particles

**vRNPs**, viral ribonucleoprotein particles

Within assembled virions, M1 forms a protein layer underneath the viral lipid envelope where it serves as an endoskeleton interacting with cytoplasmic tails of glycoproteins haemagglutinin (HA) and neuraminidase (NA) [1, 2], matrix protein 2 (M2), [3] viral ribonucleoprotein particles (vRNPs) [4, 5, 6] and the membrane [7, 8, 9, 10, 11, 12]. The critical role of M1 in assembly is exemplified by the observation that the expression of the influenza M segment genes (Matrix protein 1 and 2) together with either HA or NA is sufficient to induce assembly of filamentous particles that resemble native virions [13]. Exposure of M1 to low pH during virus entry is thought to induce a conformational change of M1 or changes in the M1 polymer arrangement, which ultimately allows disassembly and detachment from the membrane [8, 9, 14, 15]. Interactions of M1 with host cell proteins and, in particular, the nuclear transport factor transportin‐1 (TNPO1) promote its release from vRNPs [16]. As is typical for small viral proteins, M1 is thought to be a multifunctional protein, supporting multiple steps of the virus life cycle. Within infected cells, M1 can be found in the cytosol as well as in the nucleus. It is currently believed that M1, which has a nuclear localization signal (NLS), is imported into the nucleus to support nuclear export of newly formed RNPs [4, 17].

## New structures of full‐length M1

**Fig. 1:**
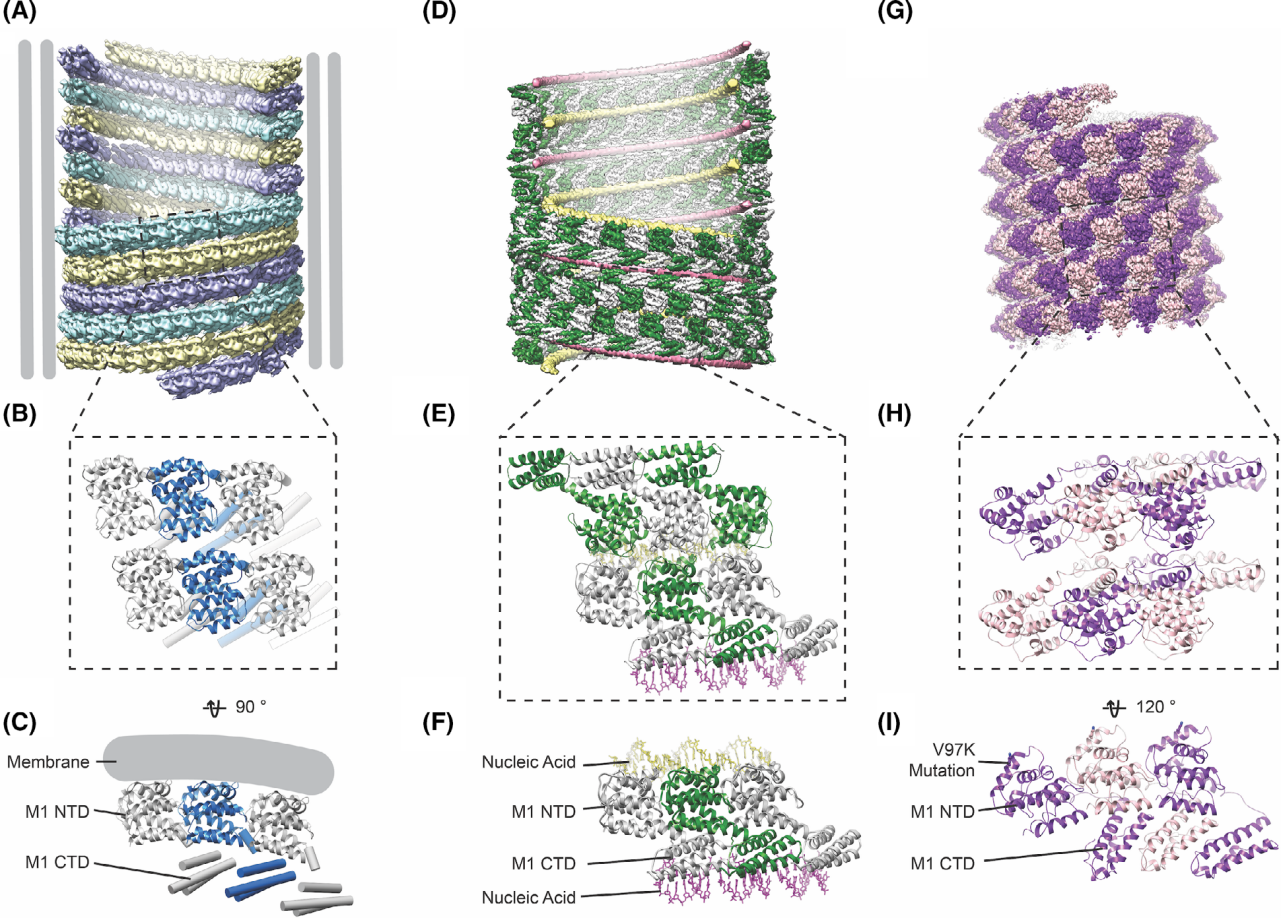
Comparison of how M1 assembles into higher‐order oligomers in the virus and *in vitro*. (A) Arrangement of M1 underneath the viral envelope, here from influenza A/Hong Kong/1/1968 (H3N2) VLPs. In the example shown here, M1 is arranged as three helical strands, each shown in a different colour (EMD‐11078) [12]. (B) Zoom into (A) illustrating the local arrangement of six M1 monomers across two neighbouring strands (PDB‐6Z5J). (C) Side view of three neighbouring monomers along a single strand indicating the relative arrangement of the NTD and the CTD and the position of the membrane. (D) Helical arrangement of nucleic‐scaffolded M1 assembled *in vitro* [12], exhibiting a left‐handed arrangement and D2 symmetry (EMD‐11079). (E) Zoom into (D) showing two neighbouring strands of M1 with three monomers each (PDB‐6Z5L). (F) Three neighbouring M1 monomers and their interactions with strands of nucleic acid *via* the M1 CTD (pink nucleic acid strand) and *via* the M1 NTD (yellow nucleic acid strand). (G) Arrangement of V97K M1 helical arrays formed *in vitro* under high‐salt conditions (EMD‐22384) [23]. (H) Zoom into (G) showing 6 monomers (PDB‐7JM3). (I) Three neighbouring monomers shown in the same orientation as (C and F) to illustrate the relative arrangement of the M1 NTD and CTD.

By using cryo‐electron tomography (cryoET) and subtomogram averaging, we were recently able to describe the structure of M1 directly within assembled influenza A/Hong Kong/1/1968 (H3N2) virions and virus‐like particles (VLPs; EMD‐11075‐11078, PDB‐6Z5J) [12]. We demonstrated that M1 oligomerizes into linear strands that coil along the inside of the lipid bilayer of filamentous virus particles in a helical arrangement (Fig. 1A). The interactions between neighbouring M1 strands appear to be flexible. This suggests an arrangement of M1 as linear polymers that are tightly packed together rather than a fixed, helically symmetric 2D array. The number of parallel M1 filaments and the handedness of the helices they form can vary between virions, as can the radius of the virions.

The C‐terminal domain (CTD) of M1 is unstructured in solution [12, 20], but within the virus it folds into a mostly alpha helical domain that binds in ‘trans’ to the N‐terminal domain (NTD) of the neighbouring M1 molecule in the linear oligomer. The positively charged face of the M1 NTD faces the viral membrane, while the M1 CTD is oriented towards the virus lumen (Fig. 1A–C). Formation of the trans‐interface between NTD and CTD of neighbouring M1 molecules represents a mechanism for M1 linear oligomerization. The addition of monomers to the growing oligomeric strand, coiling along the inner surface of the budding virion, is likely to provide the energy for protruding filamentous virions from the cell surface.

The M1 layer is flexible and variable, and M1 is a small protein at the limit of what can currently be analysed using cryoET methods. For these reasons, the resolution of the structure of the M1 layer determined *in situ* is limited to ˜ 8 Å. In order to resolve high‐resolution structural details, purified M1 protein has been assembled into arrays *in vitro* that mimic the arrangement in the virion, but are more regular. We achieved this by recombinantly expressing M1 (from strain A/Puerto Rico/8/1934 (H1N1), containing substitution K134R, that was found to improve polymerization quality and is found naturally in certain virus strains) and assembling it into helical arrays in the presence of nucleic acid, which likely acts as an assembly scaffold (Fig. 1D–F; EMD‐11079, PDB‐6Z5L) [12]. In parallel, Selzer *et al*. [23] expressed recombinant M1 from the same strain with an additional charge‐introducing V97K mutation and induced it to efficiently assemble helical arrays using high‐salt (2 M NaCl) conditions (Fig. 1G–I; EMD‐22384, PDB‐7JM3). The structures of both helical arrays were determined using cryo‐electron microscopy (cryoEM) and helical reconstruction methods to 3.8 Å and 3.4 Å resolutions, respectively. The nucleic acid scaffolded arrays are formed by symmetric M1 dimers that are arranged in a D1‐symmetric helical array around two strands of nucleic acid (Fig. 1D,E), while high salt in combination with V97K induced the formation of a polar helix (Fig. 1G–I).

## Similarities and differences between *in vitro* M1 structures

Despite the substantial differences in their assembly conditions and the differences in the overall architecture and symmetry of the helical arrays, both M1 *in vitro* arrays are built from very similar linear oligomers of M1. In both structures, the NTD is formed by nine helices highly similar to the NTD structures previously determined by X‐ray crystallography [16, 24]. In both cases, as observed *in situ* within virions, the last helix of the NTD (helix 9; H9) connects to the CTD. The CTD forms an alpha helical domain that is bound in trans to the NTD of a neighbouring M1 monomer (Fig. 1C,F,I).

**Fig. 2:**
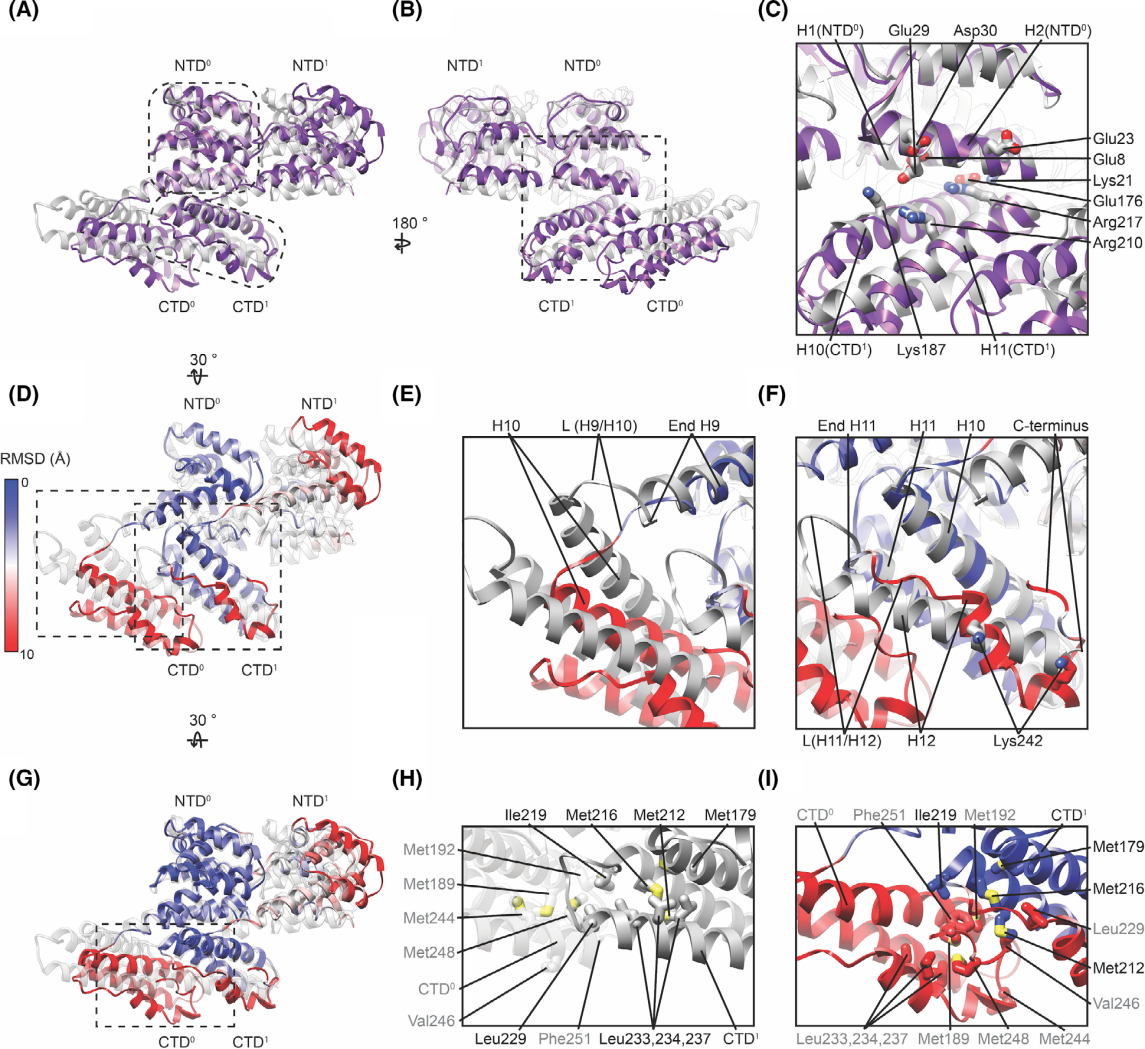
Structural similarities and differences between linear M1 oligomers from two different M1 *in vitro* arrays. (A) Overlay of two neighbouring M1 monomers as they occur in the nucleic acid scaffolded M1 array (grey, PDB‐6Z5L) and in the M1 array from V97K formed under high‐salt conditions (purple, PDB‐7JM3). The overlay was generated by aligning the NTD^0^ from both structures. (B) As (A) but rotated by 180°. The dashed outline in (A) marks the stable unit between both structures, formed by NTD^0^ and CTD^1^ from the neighbouring monomer to which it is directly bound. This unit is similar between both structures. (C) A magnified view of the interface between NTD and the neighbouring CTD indicating the presence of several charged sidechains. (D). Overlay of the two structures similar to (A) but in which the root mean squared difference (RMSD) between the two structures has been calculated and is indicated by the colour of the high‐salt assembled M1 model. This highlights the similarity between the two structures within the stable unit (blue). (E) Magnified view of the residues connecting the NTD and CTD of M1 within one monomer, where the linker L(H9/H10) is more extended in the high‐salt V97K structure (coloured by RMSD) relative to the nucleic acid scaffolded structures (grey). This leads to a change in the relative position of the CTD resulting in a higher RMSD (red). (F) Magnified view of the CTD region highlighting differences between the *in vitro* structures in H12 and the linker connecting H11 and H12. (G) As in (D) but turned by 30° to highlight interactions between CTDs of neighbouring monomers. (H) Positions of hydrophobic side chains at the interface between neighbouring CTDs in the nucleic acid scaffolded M1 array. Side chain names in grey and black are positioned on CTD^0^ and CTD^1^, respectively. (I) Positions of the same side chains shown in h marked in the high‐salt V97K structure.

Within the two different helical arrays, the linear M1 strands curve in different directions. This difference is accommodated by a movement of the CTD relative to the NTD within the same M1 monomer. This movement can be clearly seen in an overlay of the two structures (Fig. 2A–B). Despite this movement, the unit of one NTD together with its binding partner, the CTD from the neighbouring monomer, is almost identical between the two structures (Fig. 2A–D). The stable NTD/CTD interface is formed between H1 and H2 of the NTD and H10, H11 of the CTD from the neighbouring monomer and is characterized by opposing clusters of charged residues (Fig. 2C).

Although the linear oligomers of M1 are very similar in both structures, there are structural differences and these may be relevant for the various functions of the M1 protein. The superposition of the two structures reveals differences primarily at two positions: at the end of H9 where the NTD is linked to the CTD (Fig. 2E), and at the last 30 residues of the CTD including H12 (Fig. 2F).

H9 ends at residue 158 in the majority of published crystal structures of the M1 NTD [16, 24]. In both M1 *in vitro* helical assemblies, H9 is extended, by two turns in the high‐salt assembled structure, and by three turns in the nucleic acid scaffolded structure (Fig. 2D–E). The difference between the two‐ and three‐turn extensions alters the position of H10 and therefore changes the position of the CTD relative to the NTD of the same M1 monomer (Fig. 2A,D). The overall effect is therefore that the stable unit, consisting of the NTD and the neighbouring CTD to which it binds, moves slightly relative to the adjacent stable unit in the filament. It is this change that, when propagated along the linear oligomers, allows them to curve in different directions in the two different helical arrays. We speculate that structural flexibility at this position may allow the linear oligomers of M1 to adapt to the different curvatures found on the sides and at the ends of filamentous virus particles as well as the variability in curvature and helix start numbers between virions.

In the nucleic acid scaffolded helix, the last 30 residues of the CTD form H12, which is connected to H11 *via* a short loop. H12 extends downwards, away from the NTD. In contrast, in the high‐salt assembled structure, most of these last 30 residues adopt a more extended coil structure with only a short, three‐turn stretch of alpha helical structure. The final 8 amino acids in the CTD bend such that they are oriented upwards towards the NTD (Fig. 2D,F). These residues include Lys242, which is one of those mediating nucleic acid interactions in the scaffolded assembly. Presumably, the presence of nucleic acid promotes the structural differences seen in this region compared with the high‐salt assembled structure.

In both *in vitro* assemblies, methionine‐ and leucine‐rich hydrophobic clusters form the interfaces between CTDs along the M1 oligomer (Fig 2G‐I). These hydrophobic clusters encompass similar sets of residues in the two *in vitro* assemblies, but due to the altered position of the CTD relative to the NTD and the different positions of the final 30 amino acids, the arrangements of the residues within the clusters and the interfaces between monomers differ. In the nucleic acid scaffolded assembly, Met192 and Met248 on CTD^0^ form a hydrophobic interface with Ile219 and Leu229 in CTD^1^ (Fig. 2H). In the high‐salt assembly, alternative folding of the last 30 amino acids dissociates Leu233, Leu234 and Leu237 from Met212 and Met216. Met192, Val246 and Phe251 in CTD^0^ then form a hydrophobic interaction with Ile219 and the exposed Met212 and Met216 in CTD^1^ (Fig. 2I).

**Fig. 3:**
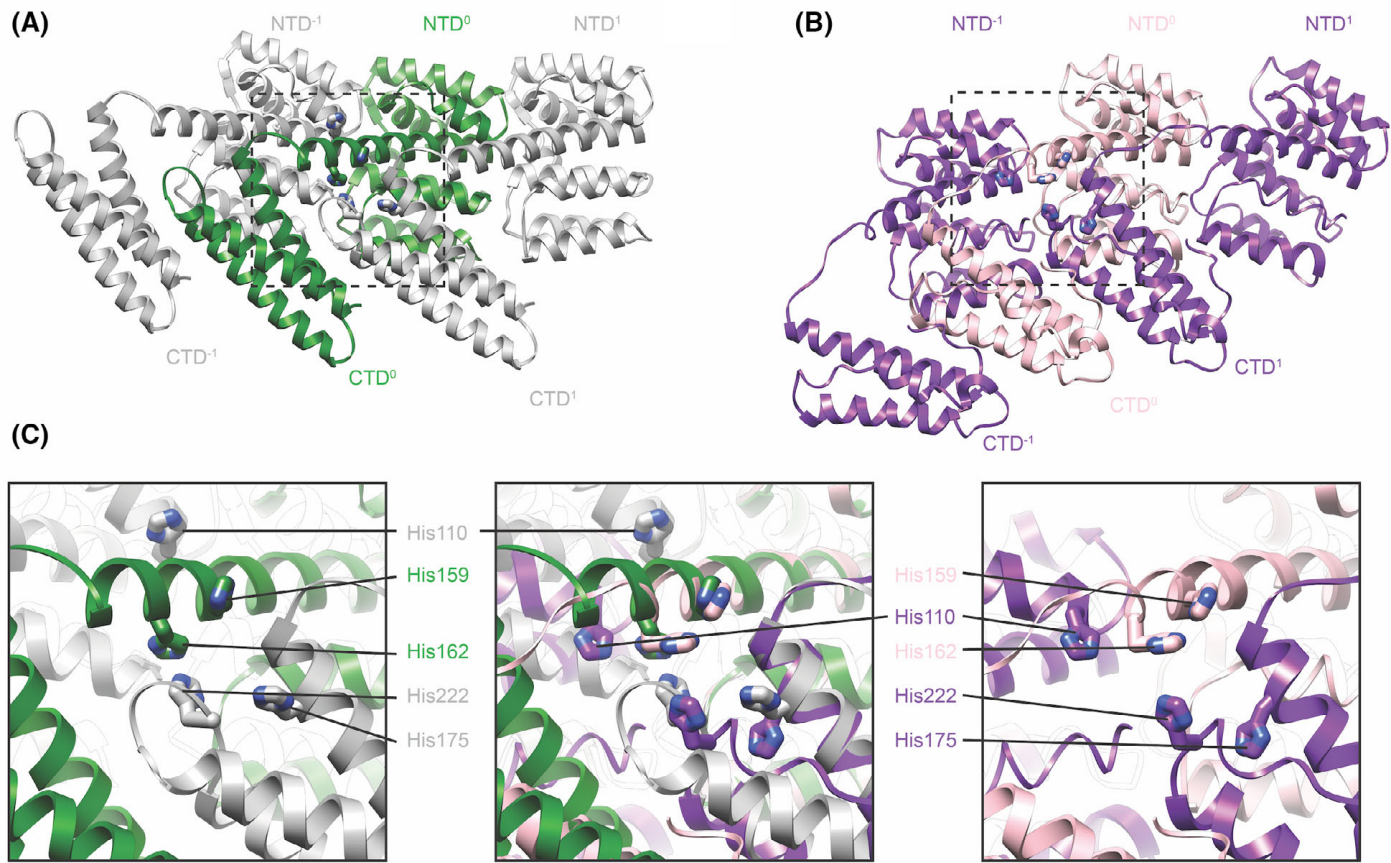
Comparison of the histidine cluster formed across three neighbouring monomers along the linear polymer. (A) Three neighbouring monomers as they occur in the nucleic acid scaffolded M1 array (PDB‐6Z5L). (B) Three neighbouring monomers as they occur in the V97K high‐salt M1 helical array (PDB‐7JM3). (C) Magnified view of the boxed region to highlight the positions of 5 histidines that form a cluster at the interface of three monomers. The left panel shows the arrangement in the nucleic acid scaffolded structure, the right panel in the V97K high‐salt structure, while the central panel shows an overlay of the two. Only the position of His110 differs significantly between the two structures.

In both *in vitro* assemblies, formation of the linear oligomer creates a cluster of 5 histidines contributed by 3 neighbouring M1 monomers. The positions of four of the five histidines are unchanged (Fig. 3), with only His110 being differently positioned due to the altered relative positions of neighbouring NTDs. Both studies proposed that this cluster may function as the pH switch, which responds to the low pH of the endosome during viral entry and triggers rearrangement of the M1 layer.

## Comparison of the *in vitro* and *in situ* M1 structures

**Fig. 4:**
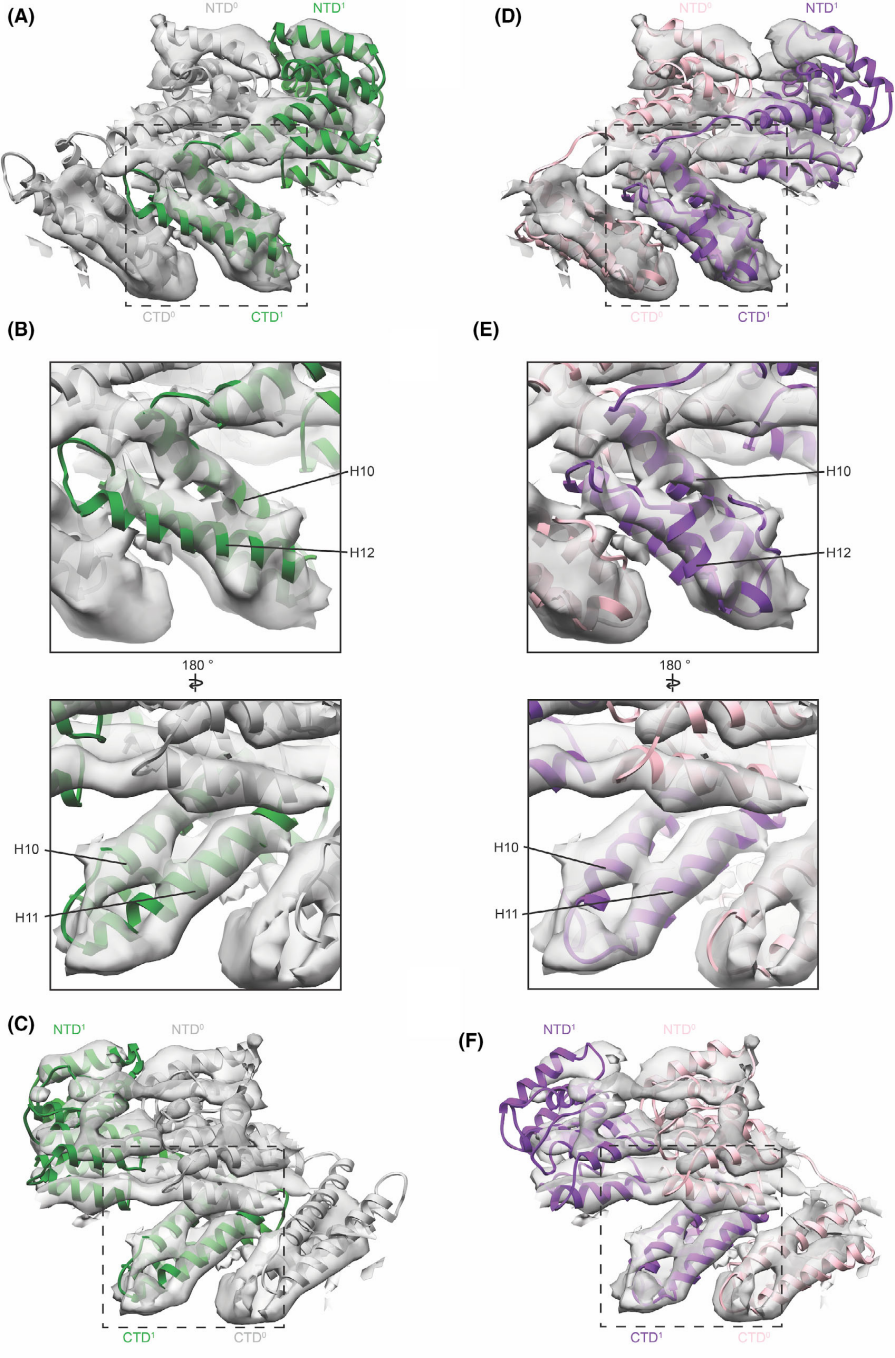
Comparison of M1 *in vitro* models to the arrangement of M1 within viruses/VLPs. (A) Two neighbouring monomers from the nucleic acid scaffolded M1 (green/grey ribbons, PDB‐6Z5L) are fitted into the structure of M1 determined *in situ* (grey isosurface, EMD‐11078). (B) Magnified views of CTD^1^ focusing on the region of H12, which is different between the two *in vitro* structures, indicating that H12 in the model is longer and rotated slightly relative to the density (left), while H10 and H11 fit the density well (right). (C) Same as (A) but rotated by 180° indicating the slight offset between model and density for CTD^0^ and NTD^1^ while the stable unit (NTD^0^ and CTD^1^) fits well. (D) Two neighbouring monomers from the V97K high‐salt M1 assembly (pink/purple ribbons, PDB‐7JM3) fitted into the M1 *in situ* structure. (E) Magnified views indicating that helix 12 and L(H11/H12) as well as H10 and H11 fit the density well. (F) Same as (D) but rotated by 180° indicating the slight offset between model and density for CTD^0^ and NTD^1^, while the stable unit (NTD^0^ and CTD^1^) fits well.

We compared the structures of the two *in vitro* M1 assemblies to the lower‐resolution structure determined from filamentous virions. Considering the similarity of the two *in vitro* structures, and the previously described similarity between the *in vitro* structures and the in‐virus structure, it is not surprising that the overall architecture of the linear M1 filaments is the same in all cases; in all cases, oligomerization involves a folded CTD interacting in trans with the neighbouring NTD (Fig. 4). However, as discussed above there are structural differences between the *in vitro* assemblies and the M1 structure found inside the virus has features of both structures. The NTD arrangement from the nucleic acid scaffolded structure is similar to the arrangement in crystals of the M1 NTD under neutral pH, [25] and this arrangement appears to best represent the NTD arrangement inside the virus [12]. On the other hand, the *in situ* density for the final 30 residues of the CTD best matches that in the high‐salt assembled *in vitro* structure where these residues bend back towards the CTD (Fig. 4D). When comparing the relative orientations of neighbouring NTD‐CTD units, we find that both *in vitro* structures match the in‐virus M1 density closely but not perfectly (Fig. 4). The relative orientation of the two domains in the virus corresponds to an intermediate between the two *in vitro* structures, and we speculate that H9 may be extended by 2.5 turns in the virus (compared with 2 turns in the high‐salt assembled structure and 3 turns in the nucleic acid scaffolded structure). This observation is consistent with our hypothesis based on the comparison of the two *in vitro* structures that the amount of H9 extension in M1 can vary to accommodate different curvatures observed across different virions.

We therefore conclude that the NTD arrangement within the linear M1 oligomer for the major population of M1 in filamentous virions is best represented by the nucleic acid bound *in vitro* assembled structure, while the structure of the M1 CTD is best represented by the CTD from the high‐salt *in vitro* assembled structure. The relative domain orientation and H9 extension likely correspond to an intermediate between the two *in vitro* structures.

## Do the structural differences reflect multifunctionality of M1?

M1 is a multifunctional protein, with different roles at different stages in the viral lifecycle, and it is therefore possible that the conformations observed in the two different *in vitro* assembled M1 oligomers represent different functional states of M1. If this is the case, which states might they represent?

The different conformations reveal sites of flexibility; changes in the NTD/NTD interface, in H9 and in the relative orientation of the NTD and CTD, allow M1 to form oligomers with different curvatures. These may accommodate small difference in curvature depending on virus diameter or the number of M1 helices, but may also accommodate different curvatures present at the spherically curved tip or at the neck of the growing virus.

It has long been suggested that M1 undergoes a change in both its conformational and oligomerization state when the virus encounters low pH in the endosome during virus entry [9, 22, 26]. Because both *in vitro* assembled structures contain a similar, conserved, ordered histidine cluster, we think that both structures are likely to exist above the pKa of histidine within the histidine cluster and therefore that both represent neutral pH conformations. M1 has been observed to form coil‐like structures when dissociated from the virus membrane after low pH exposure [9, 15, 27], and future studies may shed light on the arrangement of the histidines within such structures.

After entry, fusion and subsequent release of M1 from the viral membrane, and once in the neutral pH of the cytoplasm, it is conceivable that M1 adopts a new conformation, for example coordinating with and protecting viral RNA, although M1 needs to be detached from the vRNPs to allow their debundling and nuclear import [4, 16]. A fraction of M1 localizes to the host cell nucleus where it is thought to associate with newly assembled vRNPs to assist with their nuclear export [4, 16, 19, 28]. This process involves interactions of M1 with RNPs, likely *via* nucleoprotein (NP) but potentially also directly *via* viral RNA [5, 6, 29], the viral protein nuclear export protein (NEP also referred to as NS2) [28, 32] and possibly additional host factors such as Hsc70 [18]. Sumoylation of M1 at Lys242 (within the CTD residues, which differ in structure between the two *in vitro* assemblies) is required for RNP binding and nuclear export. [33] Once exported, binding of M1 to the vRNPs in the cytosol prevents re‐import of the vRNPs [4, 17]. It is interesting that in the nucleic acid scaffolded structure, the NLS is bound to nucleic acid, masking the NLS, which is a known mechanism to regulate nuclear import [34]. A subpopulation of M1 is known to interact with vRNPs during assembly, bundling them inside the virus and remaining associated after virus uncoating until M1 is specifically removed by low pH exposure and interactions with host cell proteins [16, 35]. All of these M1 functions are very different from those performed during assembly of the viral endoskeleton and must involve different interactions and likely different conformations of M1. One speculative function of the arrangement of the final 30 residues of the CTD observed in the nucleic acid scaffolded structure is in forming an oligomer that assists in shielding newly synthesized RNA/RNPs during transport through the host cell’s cytosol after nuclear export.

Overall, we entertain two speculative hypotheses regarding the functional relevance of the two conformations of the final 30 residues of the CTD. First, that both may represent conformations found within the virion: one, the CTD confirmation of the high‐salt assembled structure, may correspond to the one found within the M1 endoskeleton on the sides of filaments, while the other – that observed when scaffolded with nucleic acid – may represent a subpopulation of M1 within the virion, which interacts with other virion components such as the RNPs at the front tip of the filament. Second, that the nucleic acid scaffolded conformation of the CTD reflects a conformation that interacts with RNPs during nuclear or cytosolic transport. Testing these hypotheses will require further experimental data about conformational adaptability of M1 and about the different roles and interaction partners of M1 during the virus life cycle.

## Conclusions

The new structures of full‐length M1 determined *in vitro* and within the virion have provided important new insights into assembly of influenza A. They reveal NTD‐NTD, NTD‐CTD, and CTD‐CTD interactions between M1 proteins and show that the CTD can fold to interact with the NTD from another M1 monomer, leading to linear oligomerization of M1. This suggests a molecular mechanism for virus assembly: an M1 monomer binds into a growing strand, leading to folding of the CTD and creation of a site to which the next monomer can bind. Burying of a large surface area during this processive assembly may provide the energy for protrusion of the viral filament from the cell surface. The *in* *situ* structure reveals the membrane‐interacting surface of M1, while the higher‐resolution *in vitro* structures reveal a conserved histidine cluster, which may serve as the pH switch mediating M1 disassembly upon virus entry.

Although the two *in vitro* structures and the *in situ* structure are all very similar, differences in the NTD–NTD interfaces, in H9 and at the end of the CTD hint at the conformational flexibility and multifunctional nature of the M1 protein. It is clear that there is still much to be learned about M1.
